# Stingless Bees Pollination Increases Fruit Formation of Strawberry (*Fragaria* x *annanassa* Duch) and Melon (*Cucumis melo* L.)

**DOI:** 10.21315/tlsr2022.33.1.3

**Published:** 2022-03-31

**Authors:** Tri Atmowidi, Taruni S. Prawasti, Puji Rianti, Fikrunnia A. Prasojo, Nalendra B. Pradipta

**Affiliations:** Department of Biology, Faculty of Mathematics and Natural Science, Bogor Agricultural University (IPB University), Bogor, Indonesia

**Keywords:** Stingless Bees, Pollination, Greenhouse, Strawberry, Melon

## Abstract

Stingless bees (Apidae: Meliponini) are distributed in tropical and subtropical areas in the world. Stingless bees are potential pollinator to increase yield of various crops species. We measured the pollination effectiveness of stingless bees, i.e., *Tetragonula laeviceps* in strawberry (*Fragaria* x *annanassa*) and *Heterotrigona itama* in melon (*Cucumis melo*) in the greenhouse. Pollination effectiveness of stingless bees were measured based on their visiting activities, i.e., foraging rate and flower handling time using focal sampling method. Measurements of fruit set consist of the number of fruits per plant, the number of normal and abnormal fruits, and the size and weight of fruits. Results showed that visiting activity of *T. laeviceps* in strawberry flowers ranged 2.33–2.73 flowers/3 min, while *H. itama* in melon flowers ranged 1.77–7.12 flower/3 min. Peak activities of *H. itama* in melon (7.12 flowers/3 min) occurred at 9.00 a.m. to 10.00 a.m., while *T. laeviceps* in strawberry (2.73 flowers/3 min) occurred at 11.00 a.m. to 12.00 p.m. Pollination by *T. laeviceps* increased 78.9% of fruit formation and reduced 16.7% of abnormal fruits of strawberry. In melon, ratio between female and male flowers was 0.03. The number of fruits produced in melon with *T. itama* (6.1 fruits/plant) was higher than in open field (2.6 fruits/plant) and control plants (no pollination) (0.2 fruits/plant). Pollination by *H. itama* increased fruit formation of melon.

**Highlight**

- The visiting activity of *H. itama* on melon flowers was higher than *T. laeviceps* on strawberry flowers and the peak visiting activity occurred in the morning.
- Visiting activities of both species of stingless bees were positively correlated with air temperature and light intensity, but negatively correlated with humidity.
- Pollination by *Tetragonula laeviceps* increased of fruits formation and decreased of abnormal fruits of strawberries, while pollination by *H. itama* increased fruit formation of melon.

## INTRODUCTION

Stingless bees (Apidae: Meliponini) are eusocial insects which are distributed in tropical and subtropical areas around the world (Neotropical, Afrotropical and Indo-Malaya) (Michener 2007). Stingless bees consist of 600 identified species from a total of about 61 genera (Rasmussen & Cameron 2010). Forty species belonging to 10 genera of stingless bees were found in Indonesia (Kahono *et al*. 2018), 22 species in West Sumatera (Inoue *et al*. 1985) and nine species in East Kalimantan (Syafrizal *et al*. 2014).

Indo-Malaya stingless bee has a high prospects as a pollinator of agricultural crop related to its small body size, producing bee products (such as honey, bee pollen and propolis), no stinging, have a high foraging activity, easy to manage, and high adaptation to environmental stress (Kahono 2015). Stingless bees play an important role in pollinating of various plant species (Michener 2007), include in mustard (*Brassica rapa*) (Atmowidi *et al*. 2007). Previously, the use of stingless bees for pollination of agricultural plants were reported in *Jatropha curcas* (Kasno *et al*. 2010), strawberry (Widhiono *et al*. 2012), hot pepper (*Capsicum annuum*) in the farm system (Putra *et al*. 2014), chili in the green house at Malaysia (Azmi *et al*. 2016), kale (*Brassica oleracea*) (Wulandari *et al*. 2017), and cucumber (Tej *et al*. 2017). In this study, we used two species of agricultural plants, namely strawberry (*Fragaria* x *annanassa* Duch) and melon (*Cucumis melo* L.) to assess the effectiveness of pollination of stingless bees.

Strawberry is a herbaceous perennial plant in subtropics to temperate climate that successfully cultivated in wide range of climatic condition (Singh *et al*. 2006). Fruits of strawberry contains a high vitamin C and bioactive compounds, such as anthocyanins, polyphenols, tannins and flavonoids (Bhat & Stamminger 2015). This plant has hermaphrodite flowers and is generally self-fertile. Nectaries located at the base of the flower (Delaplane & Mayer 2000). The formation of strawberry fruits depends on the number of stigma per flower. Most varieties of strawberry are self-compatible and self-pollinated normally or by wind pollination. However, in the same flower, receptive of stigma occurred before the anther releases pollens, so that the process of allogamy occurs (Free 1993). Therefore, strawberry also depends on insects pollination (Zebrowska 1998).

Melon is a horticultural commodity in Indonesia and is widely grown especially in dry season. Melon fruits contains low calories and fat and also as a source of vitamin A, vitamin B complex, vitamin C, polyphenols and carotenoids (Lester 2008). In 100 g of fresh melon fruit contains 92.1% of water, 0.5% of protein, 0.3 % of fat, 6.2 % of carbohydrate, 0.5 % of fiber, and 350 IU of vitamin A (Daryono *et al*. 2019).

Melon is a monoecious plant with staminate and hermaprodhite flowers with a sex ratio 18:1. Staminate flowers consisted five yellow petals fused at the base and androecium consisted five stamens with fused anthers and filaments. While hermaprodhite flowers have a bigger and similar perianths with staminate flowers. Anthesis of staminate flowers occurred earlier in the morning than hermaprodhite flowers and both types of flowers have longevity in one day (Revanasidda & Belavadi 2019).

Melon require pollinators to transfer pollens from anther to stigma and affected to fruits and seeds formation (Aizen *et al*. 2009). In India, Revanasidda and Belavadi (2019) reported flowers of muskmelon visited by 16 species of insects, i.e., 13 species hymenopteran, two lepidopteran and one dipteran and *Apis cerana* and *Apis florea* were as dominant species. Efficiency of pollinators related to the biology and morphology of flowers as attractants, such as petal colours, aromas, nectar content, pollens, and oils (Freitas & Paxton 1998). In this study, we measured the pollination effectiveness of *T. laeviceps* in strawberry and *H. itama* in melon plants.

## MATERIALS AND METHODS

### Plants and Stingless Bees

We used strawberry var. earlibrite and melon plants in the greenhouse. Strawberry plants were located at Bandung and melon plants in Cikabayan field station of IPB University in Bogor, West Java, Indonesia. A total of 750 strawberry plants were used in this study divided into three groups: 300 plants with two colonies of *T. laeviceps*, 300 plants without bee colonies (control), and 150 plants located outside of greenhouse (open fields). We used 90 melon plants consisting of 30 plants located in the greenhouse with one colony of *H. itama*, 30 plants in the greenhouse without bee colony (control), and 30 plants located outside of a greenhouse.

### Observation of Stingless Bees Visiting Activities

Visiting activities of stingless bees on strawberry and melon flowers were observed by using focal sampling method (Dafni 1992) from 08.00 a.m. to 4.00 p.m. for 20 days in sunny days. Visiting activities observed were the number of flowers visited per 3 min (foraging rate) and the duration of visits per flower (flower handling time). Environmental parameters, like temperature, humidity, and light intensity were also measured every one hour during the observations.

### Fruits Set Measurements

Ten individuals of strawberry plants of each group were selected to measure the fruits set. Measurements of the fruit set consist of the number of fruits per plant, the number of normal and abnormal fruits, the size and weight of each fruit. In melon plants, we also measured the total number of fruits and the average number of fruits per plant.

### Data Analysis

The number of fruits, fruit size, and fruit weight of strawberry among groups were analysed using analysis of variance (ANOVA) and Tukey’s test. The number of male flowers, female flowers, and the number of fruits of melon plants among groups were analysed using Kruskal-Wallis and Mann-Whitney test using Paleontological Statistics Software (PAST) (Hammer *et al*. 2001).

## RESULTS

### Visiting Activities of Stingless Bees

**Figure 1:**
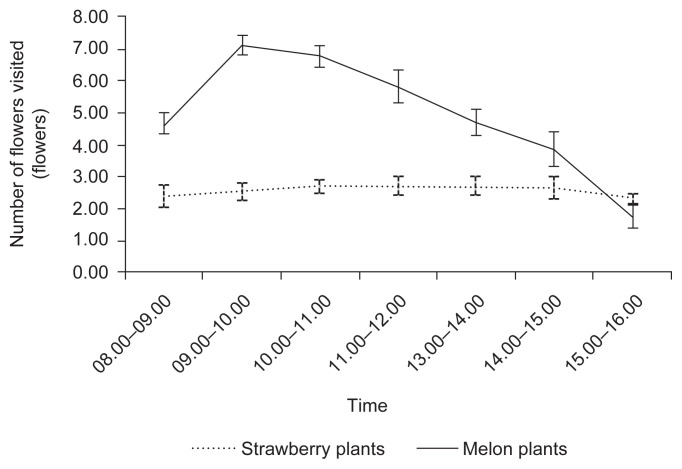
The number of flowers visited by *T. laeviceps* in strawberry flowers and *H. itama* in melon flowers per 3 min. Standard deviations are shown in the graphic.

In general, there was high visitation activity of *T. laeviceps* in strawberries flowers and *H. itama* on melon flowers. In melon plants, *H. itama* visits the flowers ranged 1.77–7.12 flowers/3 min, while *T. laeviceps* in strawberry plants ranged 2.33–2.73 flowers/3 min. The peak activity of *H. itama* on melon flowers (7.12 flowers/3 min) occurred at 09.00 a.m. to 10.00 a.m, while *T. laeviceps* in strawberry flowers (2.73 flowers/3 min) occurred in 11.00 a.m. to 12.00 a.m. Visiting activities of both species decreased in 12.00 a.m. to 4.00 p.m. (Fig. 1).

**Figure 2:**
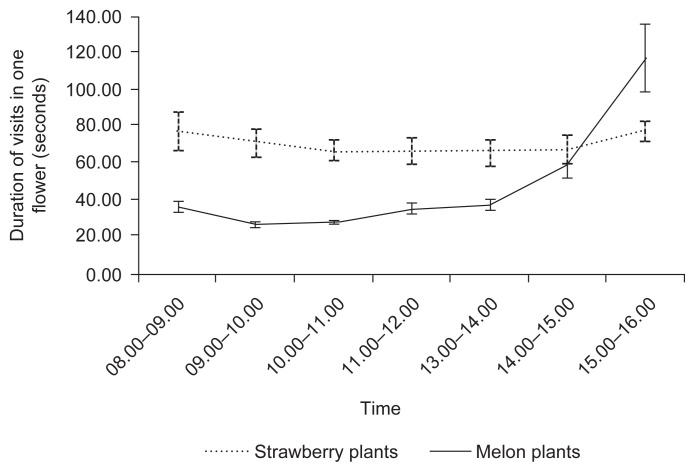
Duration of visits of *T. laeviceps* in strawberry flowers and *H. itama* in melon flowers per flower. Observations were conducted in 20 days. Standard deviations are shown in the graphic.

The higher visiting activities of stingless bees in the morning caused the duration of visits per flower was short. The duration of visits of *T. laeviceps* in strawberry ranged 66.07–77.81 sec/flower, while *H. itama* on melon plants ranged 26.43–117.65 sec/flower. The duration of visit per flower in the afternoon was relatively long for both bee species in the bioassay (Fig. 2).

The temperature, humidity, and light intensity during the bee observation in strawberry plants of West Bandung were 29.4°C, 60.6% and 1,047.4 lux, respectively. Meanwhile, the average of temperature, humidity, and light intensity in melon plants of Dramaga, Bogor were 32.4°C, 60.3% and 10,060.6 lux. Pearson correlation analysis showed that visiting activities of both species were significantly positive correlation with air temperature (*r* = 0.43, *P* = 1.04E-07; *r* = 0.25036, *P* = 2.51E-12) and light intensity (*r* = 0.25, *P* = 0.002166; *r* = 0.49892, *P* = 4.44E-49), but negatively correlated to humidity (*r* = −0.35, *P* = 1.42E-05; *r* = −0.53858, *P* = 2.19E-58) (Table 1).

### Fruits Formation

Strawberry plants pollinated by *T. laeviceps* produced more fruits (3.4 fruits/plant) compared to open fields to all pollinators visiting (1.7 fruits/plant) and without bees (1.9 fruits/plant). Pollination by *T. laeviceps* also produced more normal fruits (2.9 fruits/plant) than plants outside of the greenhouse (0.8 fruit/plant) and without bees (no pollination) (1.3 fruits/plant). *T. laeviceps* helps pollination of strawberry plants and increased 78.9% of fruits formation, 123.1% of normal fruits, and decreased 16.7% of abnormal fruits. Fruit size and weight also increased in 3.5% and 5.4%, respectively, in strawberry pollination by *T. laeviceps.* The number of abnormal fruits produced by plants with stingless bees (0.5 fruits/plant) was not different from plants without bees (control) (0.4 fruit/plants) and open visitation (0.9 fruit/plant) (ANOVA, *P* = 0.225 and *P* = 0.079) (Table 2).

In melon plants, the number of male flowers produced by 30 plants in each group were not significantly different (1,188, 1,229 and 1,298 flowers, respectively). The number of female flowers of control plants was low (total 34 flowers, average 0.2 flower/plant) and the ratio between female and male flowers was 0.03. The total number of fruits produced in plants with *T. itama* (183 fruits) was higher than plants in outside greenhouse (78 fruits) and control plants (7 fruits) (Table 3).

## DISCUSSION

### Visiting Activities of Stingless Bees

The foraging activities of *T. laeviceps* and *H. itama* related to ambient temperatures in each location (Bandung: 29.4°C and Bogor: 32.4°C). But, we proposed that temperature difference between the two observation sites did not affect to the foraging activity of the two bee species. Environmental conditions affected thermoregulation of bees (Sakagami *et al.* 1983). The activities of *T. laeviceps* on the strawberry flower of this study (2.73 flowers/3 min) was lower than in strawberry (3.4 flowers/min) (Harahap 2013) and 4.4 flowers/min on teak in Thailand (Tangmitcharoen *et al.* 2006). The peak activity of *T. laeviceps* in the current study (10.00 a.m. to 02.00 p.m.) was similar reported in North Vietnam (Chinh *et al.* 2005). In Padang, West Sumatera, Indonesia, foraging behaviour of *T. minangkabau* and *T. moorei* starting in the morning until afternoon (Inoue *et al*. 1985). Previously, reports of visiting activities of the stingless bees on melons have not been reported. Visiting activities of honey bee, *A. mellifera* on hermaphrodite and male flowers of yellow melon were reported in Brazil (Ribeiro, da Silva, *et al.* 2015; Ribeiro, Silva, *et al*. 2017).

Flight activities of insects are affected by environmental conditions. The foraging behaviour of *Heriades* sp. aff. *fulvescens* was affected by microclimate, quality of nectar and pollens (Klein *et al.* 2004). Results showed that the foraging activity of *T. laeviceps* and *H. itama* were positively correlated with temperature and light intensity and negatively correlated with humidity. Similar results also was reported that temperature, light intensity and humidity affected the distribution and abundance of *T. laeviceps* (Liow *et al.* 2001). The foraging behaviour of *A. mellifera* affected by light intensity, temperature and humidity (Anendra 2010).

### Fruits Formation

Results showed *T. laeviceps* pollination increased the number of fruits, fruits size and weight and decreased the number of abnormal fruits of strawberry. Wind and gravity pollination are not sufficient to promote an appropriate flower pollination (Albano *et al*. 2009). This results indicate stingless bee was an effective pollinator for strawberries. The use of *T. laeviceps* in pollination of strawberry in Ciwidey, South Bandung increased the number of fruits, fruits weight and vitamin C content by 40.4%, 105.9% and 7.3%, respectively (Harahap 2013). Pollination of stingless bees, *Scaptotrigona* aff. *depilis* and *Nannotrigona testaceicornis* reduced 4% of abnormal fruits of strawberry in the greenhouse and four times visiting of individuals are needed to develop well-formed fruits (Roselino *et al.* 2009). The success of strawberry pollination is based on fertilisation of the achenes (Csukasi *et al.* 2011). Allocation of pollens on receptacles, increasing the fertilised achenes of fruit (Svensson 1991) and insufficient pollination resulting unfertilised achenes that no physiological functionality (Free 1993). Achene is produced by a fertilised ovule. Achene is protected by tissue and produces auxin that stimulates receptacle to form fruit (Csukasi *et al*. 2011). In unfertilised ovules, receptacles do not develop and form abnormal or small-sized fruits (Nitsch 1950).

Stingless bee, *H. itama* plays an important role in pollen transfer of melon. The anthesis of both flowers occurred in the early morning (06.00 a.m.) and staminate flowers opened an hour earlier to hermaprodhite flowers. The stigma receptivity duration was between 08.00 a.m. to 06.00 p.m. and the peak receptivity occurred around 06.00 pm (Revanasidda & Belavadi 2019). To stimuli melon fruit formation, at least 500 viable pollens are needed on stigma (Mussen & Thorp 1997). Revanasidda and Belavadi (2019) also reported to set fruits, muskmelon required 15 to 20 bee visit/flower and there was no fruit set with 0, 1 and 2 visits/flower/day. Our visual observation also showed that foraging time of the bees coincide with the stigma receptiveness. The results showed that the number of fruits produced by melon pollinated *H. itama* was higher than open visitation and no pollinators (control plants). Stingless bees, *Scaptotrigona* aff. *depilis* and *N. testaceicornis* also increased fruit formation of cucumber and decreased abnormal fruits (Santos *et al.* 2008). In summer squash (*Cucurbita pepo*), fruit quality depends on the effectiveness of pollination and fruits production decreased when inadequate pollination (Cane *et al.* 2011). Pollinating insects increased the number of fruits and seeds set (Faegri & van der Pijl 1971).

Stingless bees are highly diverse and abundant group of eusocial bees that distributed in the tropical and subtropical areas of the world (Michener 2007). Current study showed that stingless bees, *T. laeviceps* increased fruit production of strawberry and *H. itama* increased of yields of melon. Previous study showed that stingless bees are effective and important pollinators of various crops and contribute to pollination of more than 60 commercial plant species (Heard 1999). Meliponini are generalist forager that collect nectar and pollen from various plants species (Ramalho *et al.* 1990; Biesmeijer *et al*. 2005). Stingless bees also can replace honey bee pollination due to various factors, such as a miss-match in body size and flower size, specialised pollen release mechanisms, and low nectar production of plants (Kearns & Inouye 1997). In agricultural crops, stingless bees are even more susceptible of pesticides due to smaller-body size with high surface area-to-volume ratio (Slaa *et al*. 2006). Application of pesticides should be managed to minimise the impact on Meliponini.

## CONCLUSION

The peak visiting activities of *T. laeviceps* on strawberry flowers in West Bandung occurred at 11.00 a.m. to 12.00 p.m., while *H. itama* on melon flowers in Dramaga, Bogor occurred at 09.00 a.m. to 10.00 a.m. Pollination by *T. laeviceps* on strawberry plants increased 78.9% of fruit formation, 123.1% of normal fruits and decreases 16.7% of abnormal fruits. In melon plants, pollination by *H. itama* increased fruits formation. Results showed both Indonesian species of stingless bees were effective in pollinating of strawberry and melon crops in the greenhouse.

## Figures and Tables

**Table 1:** Correlation between visiting activities of stingless bees and environmental parameters.

Environmental parameters	Strawberry		Melon	

	*r*	*P*	*r*	*P*
Temperature (°C)	0.43029	1.12E-07	0.25036	2.51E-12
Light intensity (lux)	0.24875	0.0030406	0.49892	4.44E-49
Relative humidity (%)	−0.34164	3.61E-05	−0.53858	2.19E-58

*Notes*: *r* = Pearson correlation coefficient, *P* = significance value.

**Table 2:** Fruits formation of strawberry plants with *T. laeviceps*, open fileds and control plants.

Fruits parameters	Treatments			

	Plants with *T. laeviceps*	Open fields	Control plants (no pollinators)	Increase (%*)*
Number of fruits	3.4^a^	1.7^b^	1.9^b^	78,9
Number of normal fruits	2.9^a^	0.8^b^	1.3^b^	123.1
Number of abnormal fruits	0.5^a^	0.9^a^	0.4^a^	−16.7
Fruits length (cm)	4.6^a^	4.1^a^	4.5^a^	3.5
Fruits weight (g)	9.7^a^	8.9^a^	9.2^a^	5.4

*Note*: Different letters in the same row were significantly different based on one-way ANOVA and Tukey’s test. The numbers in parentheses ( ) indicate the minimum-maximum value.

**Table 3:** Number of male and female flowers and fruits formation in melon plants with *H. itama*, open fileds, and control plants (no pollinators).

Flowers and fruits parameters	Treatments		

	Plants with *T. itama*	Open fileds	Control plants (no pollinators)
Number of male flowers (flowers)	1188^a^	1229^a^	1298^a^
Number of female flowers (flowers)	183^a^	172^a^	34^b^
Number of fruits of 30 plants (fruits)	89^a^	78^a^	7^b^
Ratio of female: male flowers	0.15	0.14	0.03
Number of fruits per plant (fruits)	3.0	2.6	0.2

*Note*: Different letters in the same row were significantly different based on Kruskal-Wallis and Mann-Whitney test.

## References

[b1-tlsr-33-1-43] Aizen MA, Garibaldi LA, Cunningham SA, Klein AM (2009). How much does agriculture depend on pollinators? Lessons from long-term trends in crop production. Annals of Botany.

[b2-tlsr-33-1-43] Albano S, Salvado E, Duarte S, Mexia A, Borges PAV (2009). Pollination effectiveness of different strawberry floral visitors in Ribatejo, Portugal: Selection of potential pollinators. Part 2. Advances in Horticultural Science.

[b3-tlsr-33-1-43] Anendra YC (2010). Aktivitas Apis cerana mencari polen, identifikasi polen dan kompetisi menggunakan sumber pakan dengcan Apis mellifera. Master’s thesis.

[b4-tlsr-33-1-43] Atmowidi T, Buchori D, Suryobroto B, Hidayat P (2007). Diversity of pollinator insect in relation to seed set of mustard (*Brassica rapa* L.; Cruciferae). Hayati Journal of Biosciences.

[b5-tlsr-33-1-43] Azmi WA, Seng CT, Solihin NS (2016). Pollination efficiency of the stingless bee, *Heterotrigona itama* (Hymenoptera: Apidae) on chili (*Capsicum annuum*) in greenhouse. Journal of Tropical Plant Physiology.

[b6-tlsr-33-1-43] Biesmeijer JC, Slaa EJ, Siqueira de Castro M, Viana BF, Kleinert A, Imperatriz-Fonseca VL (2005). Connectance of Brazilian social bee-food plant networks is influenced by habitat, but not by latitude, altitude or network size. Biota Neotropica.

[b7-tlsr-33-1-43] Bhat R, Stamminger R (2015). Preserving strawberry quality by employing novel food preservation and processing techniques – recent updates and future scope – an overview. Journal of Food Process Engineering.

[b8-tlsr-33-1-43] Cane JH, Sampson BJ, Miller SA (2011). Pollination value of male bees: The specialist bee *Peponapis pruinosa* (Apidae) at summer squash (*Cucurbita pepo*). Environmental Entomology.

[b9-tlsr-33-1-43] Chinh TX, Sommeijer MJ, Boot WJ, Michener CD (2005). Nest architecture and colony characteristics of three stingless bees in North Vietnam with the first description of the nest of *Lisotrigona carpenteri* (Hymenoptera: Apidae, Meliponini). Journal of the Kansas Entomological Society.

[b10-tlsr-33-1-43] Csukasi F, Osorio S, Gutierrez JR, Kitamura J, Giavalisco P, Nakajima M, Fernie AR, Rathjen JP, Botella MA, Valpuesta V, Medina-Escobar N (2011). Gibbellerin biosynthesis and signalling during developement of the strawberry receptacle. New Phytologist.

[b11-tlsr-33-1-43] Dafni A (1992). Pollination ecology: A practical approach.

[b12-tlsr-33-1-43] Daryono BS, Subiastuti AS, Fatmadanni A, Sartika D (2019). Phenotypic and genetic stability of new Indonesian melon cultivar (*Cucumis melo* L. ‘Melonia’) based on ISSR markers. Biodiversitas.

[b13-tlsr-33-1-43] Delaplane KS, Mayer DF (2000). Crop pollination by bees.

[b14-tlsr-33-1-43] Faegri K, van der Pijl L (1971). The principles of pollination ecology.

[b15-tlsr-33-1-43] Free JB (1993). Insect pollination of crops.

[b16-tlsr-33-1-43] Freitas BM, Paxton RJ (1998). A comparison of two pollinators: The introduced honeybee *Apis mellifera* and an indigeneous bee *Centris tarsata* on cashew *Anacardium occcidenale* in its native range of NE Brazil. Journal of Applied Ecology.

[b17-tlsr-33-1-43] Hammer O, Harper DAT, Ryan PD (2001). Past: Paleontological statistics software package for education and data analysis. Palaeontologia Electronica.

[b18-tlsr-33-1-43] Harahap KK (2013). Efektivitas polinasi Apis cerana Fabricus dan Trigona laeviceps Smith (Hymenoptera: Apidae) pada Fragaria x ananassa kultivar earlibrite. Master’s thesis.

[b19-tlsr-33-1-43] Heard TA (1999). The role of stingless bees in crop pollination. Annual Review of Entomology.

[b20-tlsr-33-1-43] Inoue T, Salmah S, Abbas I, Yusuf E (1985). Foraging behaviour of individual workers and foraging dynamics of colonies of three Sumatran stingless bees. Researches on Population Ecology.

[b21-tlsr-33-1-43] Kahono S (2015). Pengembangan model perlebahan LIPI untuk edukasi, ekoturisme, dan produksi yang dapat diimplementasikan kepada masyarakat (Research Report).

[b22-tlsr-33-1-43] Kahono S, Chantawannakul P, Engel MS, Chantawannakul P, Williams G, Neumann P (2018). Social bees and the current status of beekeeping in Indonesia. Asian beekeeping in the 21st century.

[b23-tlsr-33-1-43] Kasno, Hasan ZAE, Efendi DS, Syaefuddin (2010). Efektifitas tiga spesies lebah madu sebagai agen polinasi untuk meningkatkan produktivitas (>40%) biji jarak pagar (*Jatropha curcas*) pada ekosistem iklim basah. Jurnal Ilmu Pertanian Indonesia.

[b24-tlsr-33-1-43] Kearns CA, Inouye DW (1997). Techniques for pollination biologists.

[b25-tlsr-33-1-43] Klein AM, Dewenter AS, Tscharntke T (2004). Foraging trip duration and density of megachilid bees, eumenid wasps and pompilid wasps in tropical agroforestry systems. Journal of Animal Ecology.

[b26-tlsr-33-1-43] Lester GE (2008). Antioxidant, sugar, minerals and phytonutrient concentrations across edible fruit tissues of orange-fleshed honeydew melon (*Cucumis melo* L.). Journal of Agriculture Food Chemicals.

[b27-tlsr-33-1-43] Liow LH, Sodhi NS, Elmqvist T (2001). Bee diversity along a disturbance gradient in tropical lowland forests of South-east Asia. Journal of Applied Ecology.

[b28-tlsr-33-1-43] Michener CD (2007). The bees of the world.

[b29-tlsr-33-1-43] Mussen CE, Thorp RW (1997). Honey bee pollination of cantaloupe, cucumber, and watermelon.

[b30-tlsr-33-1-43] Nitsch JP (1950). Growth and morphogenesis of strawberries as related to auxin. American Journal of Botany.

[b31-tlsr-33-1-43] Putra RE, Permana AD, Kinasih I (2014). Application of asiatic honey bees (*Apis cerana*) and stingless bees (*Trigona laeviceps*) as pollinator agents of hot pepper (*Capsicum annuum* L.) at local Indonesia farm system. Psyche.

[b32-tlsr-33-1-43] Ramalho M, Kleinert-Giovannini A, Imperatriz-Fonseca VL (1990). Important bee plants for stingless bees (*Melipona* and Trigonini) and africanised honeybees (*Apis mellifera*) in neotropical habitats: A review. Apidologie.

[b33-tlsr-33-1-43] Rasmussen C, Cameron SA (2010). Global stingless bee phylogeny supports ancient divergence, vicariance, and long-distance dispersal. Biological Journal of Linnean Society.

[b34-tlsr-33-1-43] Revanasidda, Belavadi VV (2019). Floral biology and pollination in *Cucumis melo* L.: A tropical andromonoecious cucurbit. Journal of Asia-Pacific Entomology.

[b35-tlsr-33-1-43] Ribeiro MF, da Silva EMS, Júnior IOL, Kiill LHP (2015). Honey bees (*Apis mellifera*) visiting flowers of yellow melon (*Cucumis melo*) using different number of hives. Ciência Rural, Santa Maria.

[b36-tlsr-33-1-43] Ribeiro MF, Silva EMS, Kiill LHP, Siqueira KMM, Silva MP, Coelho MS (2017). Foraging of honeybees (*Apis mellifera*) on flowers of yellow melon (*Cucumis melo*): Duration of visits. Journal of Agricultural Science.

[b37-tlsr-33-1-43] Roselino AC, Santos SB, Hrncir M, Bego LR (2009). Differences between the quality of strawberries (*Fragaria* x *ananassa*) pollinated by the stingless bees *Scaptotrigona* aff. *depilis* and *Nannotrigona testaceicornis*. Genetics Molecular Research.

[b38-tlsr-33-1-43] Sakagami SF, Inoue T, Yamane S, Salmah S (1983). Nests architecture and colony composition of the sumatran stingless bees *Trigona (Tetragonula) laeviceps*. Kontyû.

[b39-tlsr-33-1-43] Singh R, Asrey R, Kumar S (2006). Effect of plastic tunnel and mulching on growth and yield of strawberry. Indian Journal of Horticulture.

[b40-tlsr-33-1-43] Slaa EJ, Sanchez LA, Sampaio MB, Hofstede FE (2006). Stingless bees in applied pollination: Practice and perspectives. Apidologie.

[b41-tlsr-33-1-43] Syafrizal, Tarigan D, Yusuf R (2014). Keragaman dan habitat lebah *Trigona* pada hutan sekunder tropis basah di hutan pendidikan Lempake, Samarinda, Kalimantan Timur. Jurnal Teknologi Pertanian.

[b42-tlsr-33-1-43] Santos SABD, Roselino AC, Bego LR (2008). Pollination of cucumber, *Cucumis sativus* L. (Cucurbitales: Cucurbitaceae) by the stingless bees *Scaptotrigona* aff. *Depilis*. and *Nannotrigona testaceicornis* Lepeletier (Hymnoptera: Meliponini) in greenhouses neotropical. Entomology.

[b43-tlsr-33-1-43] Svensson B (1991). The importance of honeybee-pollination for the quality and quantity of strawberries (*Fragaria x ananassa*) in Central Schweden. Acta Horticulturae.

[b44-tlsr-33-1-43] Tangmitcharoen SA, Takaso TB, Siripatanadilox SC, Tasen WC, Owens JN (2006). Behavior of major insect pollinators of teak (*Tectona grandis* L.): A comprasion of clonal seed orchard versus wild trees. Forest Ecology and Management.

[b45-tlsr-33-1-43] Tej K, Srinivasan MR, Rajashree V, Thakur RK (2017). Stingless bee, *Tetragonula iridipennis* Smith for pollination of greenhouse cucumber. Journal of Entomology and Zoology Studies.

[b46-tlsr-33-1-43] Widhiono IMZ, Sudiana E, Sucianto ET (2012). Potensi lebah lokal dalam peningkatan produksi buah strawberry (*Fragaria x ananassa*). Jurnal Inovasi.

[b47-tlsr-33-1-43] Wulandari AP, Atmowidi T, Kahono S (2017). Peranan lebah *Trigona laeviceps* (Hymenoptera: Apidae) dalam produksi biji kailan (*Brassica oleracea* var. *alboglabra*). Jurnal Agronomi Indonesia.

[b48-tlsr-33-1-43] Zebrowska J (1998). Influence of pollination modes on yield components in strawberry (*Fragaria x ananassa* Duch.). Plant Breeding.

